# Deciphering transcriptome profiles of peripheral blood mononuclear cells in response to PRRSV vaccination in pigs

**DOI:** 10.1186/s12864-016-2849-1

**Published:** 2016-08-15

**Authors:** Md Aminul Islam, Christine Große-Brinkhaus, Maren Julia Pröll, Muhammad Jasim Uddin, Sharmin Aqter Rony, Dawit Tesfaye, Ernst Tholen, Michael Hölker, Karl Schellander, Christiane Neuhoff

**Affiliations:** 1 Department of Animal Breeding and Husbandry, Institute of Animal Science, University of Bonn, Endenicher Allee 15, 53115 Bonn, Germany; 2 Department of Medicine, Faculty of Veterinary Science, Bangladesh Agricultural University, Mymensingh, 2202 Bangladesh; 3 Teaching and Research Station on Frankenfrost, Faculty of Agriculture, University of Bonn, Königswinter, Germany

**Keywords:** Pig, PRRSV vaccine, PBMCs, Innate immunity, Microarray, Antibody

## Abstract

**Background:**

Porcine reproductive and respiratory syndrome (PRRS) is one of the most economically important viral diseases affecting swine industry worldwide. Despite routine farm vaccination, effective control strategies for PRRS remained elusive which underscores the need for in-depth studies to gain insight into the host immune response to vaccines. The current study aimed to investigate transcriptional responses to PRRS Virus (PRRSV) vaccine in the peripheral blood mononuclear cells (PBMCs) within 3 days following vaccination in German Landrace pigs.

**Results:**

Transcriptome profiling of PBMCs from PRRSV vaccinated and age-matched unvaccinated pigs at right before (0 h), and at 6, 24 and 72 h after PRRSV vaccination was performed using the Affymetrix gene chip porcine gene 1.0 st array. Comparison of PBMCs transcriptome profiles between vaccinated and unvaccinated pigs revealed a distinct host innate immune transcriptional response to PRRSV vaccine. There was a significant temporal variation in transcriptional responses of PRRSV vaccine in PBMCs accounting 542, 2,263 and 357 differentially expressed genes (DEGs) at 6, 24 and 72 h post vaccination, respectively compared to the time point before vaccination (controls). Gene ontology analysis revealed the involvement of these DEGs in various biological process including innate immune response, signal transduction, positive regulation of MAP kinase activity, TRIF-dependent toll-like receptor signaling pathway, T cell differentiation and apoptosis. Immune response specific pathways such as cytokine-cytokine receptor interaction, chemokine signaling pathway, signal transduction, JAK-STAT pathway and regulation, TRAF6 mediated induction of NF-kB and MAPK, the NLRP3 inflammasome, endocytosis and interferon signaling were under regulation during the early stage of PRRSV vaccination. Network enrichment analysis revealed *APP, TRAF6, PIN1, FOS, CTNNB1, TNFAIP3, TIP1, CDKN1, SIRT1, ESR1* and *HDAC5* as the highly interconnected hubs of the functional network of PRRSV vaccine induced transcriptome changes in PBMCs.

**Conclusions:**

This study showed that a massive gene expression change occurred in PBMCs following PRRSV vaccination in German Landrace pigs. Within first 3 days of vaccine exposure, the highest transcript abundance was observed at 24 h after vaccination compared to that of control. Results of this study suggest that *APP, TRAF6, PIN1, FOS, CDKN1A* and *TNFAIP3* could be considered as potential candidate genes for PRRSV vaccine responsiveness.

**Electronic supplementary material:**

The online version of this article (doi:10.1186/s12864-016-2849-1) contains supplementary material, which is available to authorized users.

## Background

Porcine reproductive and respiratory syndrome (PRRS) is an emerging viral infectious disease; clinically characterized by reproductive failures in breeding sows and respiratory disorders in growing pigs [1]. PRRS causes huge economic loss, and is of major concern as animal welfare issue in swine industry worldwide [2, 3]. The disease is caused by an enveloped, positive-sense, single-stranded RNA virus called porcine reproductive and respiratory syndrome virus (PRRSV). The PRRSV, a member of arterivirus group under the family arteriviridae, is divided into two distinct genotypes namely European and North American types [4]. The PRRSV genome is approximately 15 kb containing ten open reading frames (ORF) encoded with seven structural and 14 nonstructural proteins [5, 6]. The virulent PRRSV primarily infects pulmonary alveolar macrophages, and destroy infected cells through cytopathic replication. The host-virus interaction results in a deficient host’s innate immune response indicated by a poor induction of type I interferon (IFN α/β), the potent antiviral immune responsive cytokines [7, 8]. Some of the non-structural proteins (Nsp1, Nsp2, Nsp11) and a structural protein (N protein) of PRRSV are known to be associated with IFN suppression in the infected cells [4]. The RIG-I/MDA5 and JAK-STAT pathways are two major signaling pathways for IFN production which are found to be impaired by PRRSV during acute infection [4]. Overall, the timing and the potency of the host cellular and immunological events that occur following infection are likely potential determinants governing the pathogenesis [9].

Vaccination with modified live virus has been widely practiced in the commercial swine herd as one of the cost-effective control approaches for PRRS. The live attenuated PRRSV vaccine provides sufficient protection against homologous virus but limited protection against reinfection of genetically variant strains [10]. Live viral vaccines can efficiently trigger the activation of the host immune system through evolutionarily conserved pathogen associated molecular patterns (PAMPs) allowing their recognition by pattern recognition receptors (PPRs) of immune cells [11]. Following administration, vaccine antigen produces a ‘danger signals’ which activate the monocytes and dendritic cells in such a way to secrete proinflammatory cytokines and chemokines [12]. These cytokines and chemokines lead the extravasation and attraction of monocytes, granulocytes and natural killer cells, and generate an inflammatory microenvironment, in which monocytes differentiate into macrophages, and immature dendritic cells become mature [13]. Through changing the surface receptors, macrophages and mature dendritic cells migrate towards the draining lymph nodes and induce the activation of T and B lymphocytes. T lymphocytes have two subsets namely CD4+ and CD8+ T cell. The generation and maintenance of both B cell and CD8+ T cell responses are supported by growth factors and signals provided by CD4+ T helper lymphocytes 1 and 2 (Th1 and Th2). Th1 and Th2 are controlled by regulatory T cells (Treg) involved in maintaining the immune tolerance [14]. The peripheral blood mononuclear cells (PBMCs) are the population of immune cells which includes lymphocytes (T cell, B cell and NK cells), monocytes and dendritic cells. Altogether, they play a central role in immune system against virus infection. Therefore, deciphering the PRRSV vaccine induced global transcriptome changes in PBMCs might lead to identify the molecules and signaling pathways associated with host immune responses.

The innate immune response against viruses like PRRSV is critical as such virus is continuously changing their antigenic epitopes [15]. Innate immune response is the first line defense mechanism of host cells against foreign antigen which typically occurs within hours in a non-specific manner and may persists for few days [15]. The innate immune system recruits effector cells upon antigen exposure which secret cytokines, chemokines and proteins and subsequently activate the adaptive immune system [16]. By that means, the innate immune response acts as precursor to initiate the adaptive immunity against a specific pathogen [17]. Innate immune traits have been considered as potential selection goals for disease resistance in pig breeding as innate immunity is likely to provide a common protection mechanism against multiple pathogens [18]. Genome-wide association study revealed a major quantitative trait locus (QTL) on chromosome 4 (SSC4) associated with host resistance to in-vivo PRRSV challenge [19]. The association of this region on SSC4 with PRRS resistance was further validated by the presence of single nucleotide polymorphism (SNP) marker, WUR10000125 (WUR) in the same region [20, 21]. Candidate genes in this locus on SSC4 include the interferon induced guanylate-binding protein gene family which are functionally linked to the innate immunity [22]. Therefore, genes and molecular pathways associated with improved innate immune response to PRRSV vaccine could possibly be implemented in breeding program for PRRS resistant pigs [18]. To this end, key molecules regulating the transcriptional network of PRRSV vaccine induced innate immune response in peripheral blood are highly sought.

PBMCs are the primary immune cells in blood [23] and have suitably been used for the evaluation of vaccine induced global gene expression changes for several diseases in human and non-human primates (reviewed by [24]). The porcine PBMCs have also been used for microarray analysis of immune response genes following in-vitro lipopolysaccharide stimulation [25], in-vivo mycoplasma vaccination [26], and tetanus toxoid vaccination [27]. Transcriptional responses to natural as well as experimental infection of PRRSV have been studied through global gene expression profiling of pulmonary alveolar macrophages in pig [28, 29]. However, little is known about the global transcriptome alterations in peripheral blood after PRRSV vaccination in pigs. Therefore, the aim of this study was firstly to investigate the transcriptional response to PRRSV vaccine in PBMCs of vaccinated pigs compared to unvaccinated one. Secondly to characterize the temporal patterns of global gene expression changes in PBMCs over the first 3 days following PRRSV vaccination.

## Methods

### Study design and blood sampling

A total of 12 German Landrace female piglets were housed in the pig research farm at Frankenforst, University of Bonn, Germany. Piglets were selected from two sows farrowed at the same day; all piglets were clinically healthy with no history of respiratory diseases and birth defects. After weaning, experimental piglets were divided into two groups: six in vaccinated and six in unvaccinated group. The piglets of vaccinated group were vaccinated with a modified live PRRSV vaccine of European strain (Porcilis® PRRS, MSD Animal Health, Germany) with primary dose at day 28 and booster dose at day 56 of their age according to the routine farm vaccination program. The unvaccinated group was maintained for health control without vaccine treatment. About 8 mL whole blood samples with 1.5 mL anticoagulant (0.5 M EDTA) were collected at different ages and time points before and after vaccination from pigs of both groups (Additional file 1). The blood samples collected at day 7 of age from all piglets were used for screening the PRRSV-specific maternally derived antibody response. The blood samples collected at four time points (0 (before vaccination), 6, 24 and 72 h post vaccination (hpv)) following primary vaccination from both groups (except 0 h in unvaccinated group) were used for microarray hybridization. Three individual biological replicates from both groups were selected based on their RNA quality for the microarray experiment. In addition, the blood samples from all piglets collected just before, and two weeks post primary vaccination as well as just before, and 2 weeks post booster vaccination were used for monitoring the vaccine induced antibody response by ELISA (Additional file 1).

### Isolation of PBMCs and plasma

PBMCs and plasma were separated from the whole blood sample through density gradient centrifugation (1500 rpm for 25 min) with Histopaque®-1077 (Sigma-Aldrich, Munich, Germany) according to the protocol described by Uddin et al. [30]. The PBMCs were washed three times (pelleted at 1000 rpm for 5 min) using phosphate-buffered saline with purity of ˃99 % determined by Wright-Giemsa staining.

### Measurement of plasma antibody level

To monitor the PRRSV-specific antibody titre, the plasma samples from all study animals collected at day 7, 28, 42, 56 and 70 of their age ((Additional file 1) were screened by ELISA (PRRSV-AK screening, Synlab Vet GmbH, Augsburg, Germany) according to manufacturer’s protocol. The optical density (OD) of each well was measured at 650 nm using the Bio-Rad 680 microplate reader. The presence or absence of PRRSV antibody was determined by calculating the sample to positive (S/P) ratio. The S/P ratio was calculated according to the following equation: S/P ratio (%) = 100 × [(OD of test sample – Mean OD of negative controls)/(Mean OD of positive controls – Mean OD of negative controls)]. The samples were considered to be positive for PRRSV antibody if the S/P ratio was more than 0.4 as described by Kittawornrat et al. [31].

### Isolation and quality control of total RNA

The total RNA was extracted from PBMCs using the miRNeasy mini kit (P/N 217004, Qiagen, Hilden, Germany) according to the manufacturer’s protocol along with DNase treatment (P/N 79254, Qiagen, Hilden, Germany). RNA concentration and purity were measured by NanoDrop® spectrophotometry (ND-8000; NanoDrop Technologies). RNA integrity was checked by micro capillary electrophoresis on an Agilent 2100 Bioanalyser with RNA 6000 Nanochip kit (Agilent Technologies, Waghäusel - Wiesental, Germany).

### Microarray target preparation and hybridization

To prepare the target probes of 21 microarray, about 100 ng of total RNA samples from each of seven selected time points were processed with the GeneChip® WT PLUS Reagent kit (P/N 902281; Affymetrix Inc., Santa Clara, CA, USA) according to the manufacturer’s protocol. In brief, the total RNA was subjected to synthesize the first-strand cDNA containing a T7 promoter sequence at the 5′ end followed by synthesis of the second-strand cDNA by DNA polymerase in the presence of RNase H. This double-strand cDNA was subjected to in-vitro transcription with T7 RNA polymerase for synthesis of the antisense RNA (complementary RNA, cRNA). The cRNA preparation was then purified using purification beads to improve its stability. From 15 μg of purified cRNA, the sense-strand cDNA (ss-cDNA) was synthesized by reverse transcription using random primers. The ss-cDNA contained dUTP at a fixed ratio relative to dTTP and the remaining cRNA was degraded by RNase H. After purification and quantification, 5.5 μg of ss-cDNA in a 31.2 μL volume was fragmented by uracil-DNA glycosylase (UDG) and apyrimidinic endonuclease 1 (APE 1) at the unnatural dUTP residues and breaks the DNA strand. The fragmented ss-cDNA was then labeled by terminal deoxynucleotidyl transferase (TdT) using the Affymetrix proprietary labeling reagent that is covalently linked to biotin. The hybridization of microarray probes followed by washing and staining was performed with the GeneChip® Hybridization, Wash and Stain kit (P/N 900720, Affymetrix Inc., Santa Clara, CA). For hybridization, about 130 μL hybridization cocktail containing about 3.5 μg of biotinylated ss-cDNA probes was injected into the GeneChip® Porcine Gene 1.0 ST array strip of 81/4 format (P/N 901976, Affymetrix Inc., Santa Clara, CA, USA) and incubated for 16 h in a hybridization oven (GeneChip® Hybridization oven 640; Affymetrix Inc.) at 45 °C with 60 rpm. The hybridized chips were stained and washed in a fluid station (GeneChip® Fluidics Station 450; Affymetrix Inc.) and scanned by Affymetrix GeneChip® scanner 3000 7G. The Affymetrix GeneChip® Command Console™ (AGCC) software was used to evaluate the array images and to export the reports of spot intensity data in CEL file format.

### Microarray data processing

Pre-processing, normalization and statistical analyses of microarray dataset were performed using packages of Bioconductor-platform implemented in R-project software (v3.1.2) [32]. The ‘oligo’ package was implemented for the RMA (Robust Multi-array Average) based quantile normalization of microarray data at transcript level [33]. For quality control, some diagnostic plots of the raw intensity data were checked before and after the normalization. After excluding two arrays at 72 h post unvaccinated sample which did not pass the quality control, 19 arrays were used for further analysis. After normalization, the main probes (19,218) of the array were extracted. Then interquartile range (IQR) based filtering (variance cutoff value 0.25) was applied which further excluded about 4,978 low expressed probes. Finally the expression dataset comprising 14,231 transcript probes were subjected for downstream analysis. Probe to gene transcript annotation was performed with recent Affymetrix annotation file for assigned array [34]. Gene annotations were extended by their orthologous human gene symbol as well as ensembl gene identifiers. Until otherwise mentioned, downstream functional analyses of this dataset were performed based on human genome database.

### Characterization of phenotypic groups

To characterize the differences of transcriptional responses between pigs of vaccinated and unvaccinated group, the annotated gene expression profiles of PBMCs were subjected to an exploratory functional analysis through gene set enrichment analysis (GSEA) algorithm implemented in GSEA-P tool [35]. Two pairs of vaccination-time point group (6 hp vaccinated vs. 6 hp unvaccinated and 24 hp vaccinated vs. 24 hp unvaccinated) were considered as input phenotype for this analysis. The normalized and filtered expression dataset of 12 arrays containing human orthologous symbols of gene transcripts with their corresponding expression values were uploaded into the GSEA-P to generate the list of ranked order gene markers. The ‘immunologic signature’ catalog of gene set from Molecular Signatures Database (C7: MSigDB v5.0, Broad Institute, Cambridge, MA) was screened against the ranked gene list. The normalized enrichment score (NES) of each gene set was estimated by the number of over-representation of members of gene set towards the top or bottom of the ranked gene list through applying a weighted Kolmogrov-Smirnov statistics [36]. Then the enrichment score *p-*values were estimated using a phenotype based permutation test procedure. The statistical significance was defined by the cutoff value of false discovery rate (FDR) <0.15 and the NES *p* < 0.05.

### Differential gene expression analysis

To explore the temporal variation of transcriptional response to vaccination, differential gene expression analysis was performed using the linear analysis of microarray technique from the ‘limma’ package [37] with empirical Bayes adjustment to the variance, followed by Benjamini and Hochberg (BH) correction for multiple testing [38]. To check whether there was temporal variation among the pigs of unvaccinated control group, two contrast pairs (i.e. 0h_vac vs. 6h_unvac and 0h_vac vs. 24h_unvac) were tested. Then within the vaccinated group, three pairwise comparisons (6 hpv vs. control; 24 hpv vs. control and 72 hpv vs. control) were taken in to account for the differential expression analysis. Gene transcripts were considered as differentially expressed when passing the thresholds of false discovery rate (FDR) of <0.01 and log_2_ fold-change either >1.5 or < −1.5. The number of differentially expressed genes in each contrast pair and their interaction were exported in intersecting Venn diagram.

### Gene ontology and pathway analysis

For biological interpretation of the transcriptome dataset, the significantly over-represented gene ontology terms and biological pathways were explored with the InnateDB pathway analysis tool [39]. First, the identifiers of DEGs from microarray data were converted to their human ensembl orthologues using the BioDBnet tool (http://biodbnet.abcc.ncifcrf.gov/). The list of ensembl gene identifiers was then uploaded in InnateDB web and performed the over-representation analysis with implementation of the hypergeometric algorithm and the Benjamini-Hochberg (BH) multiple test correction method. The gene ontology (GO) and pathways were considered significantly over-represented if they had a FDR < 0.05.

### Sub-network enrichment analysis

To visualize the PRRSV vaccine induced transcriptional network as well as to identify the regulatory genes, the sub-network analysis was performed using NetworkAnlayst online tool [40]. This tool uses the InnateDB (downloaded June 20, 2014) protein-protein interaction (PPI) datasets comprised of 14,755 proteins and 145,955 experimentally validated interactions for human. NetworkAnlayst implements the R package ‘igraph’ for network analysis and ‘Gephi Toolkit’ for finalizing the network layout. Human orthologous ensembl gene identifiers of the DEGs were uploaded into the NetworkAnlayst to construct the interacting network. First, a default network was assembled based on the Walktrap algorithm taking only direct interaction of seed genes (first-order interactors). The network size was then adjusted for <500 seeds and 200 ~ 2000 nodes using the ‘reduce’ panel for high-performance visualization. Two topological measures such as degree (number of connections to other nodes) and betweenness centrality (number of shortest paths going through the node) were taken in to account for detecting highly interconnected hubs of the network. Centrality measures of hub nodes were evaluated serially with degree followed by betweenness. Nodes having higher degree and betweenness values were considered as potentially important network hubs in cellular signal trafficking. Finally, weighted network based module detection was perform to cluster the genes of similar biological functions. The *p* value of a given network module was calculated using a Wilcoxon rank-sum test of the “internal” (edges within in a module) and “external” (edges connecting the nodes of other modules) degrees. The p values were calculated based on their connectivity assuming null hypothesis that there is no difference between the number of “internal” and “external” connections to a particular node in the module. Module having more internal than external edges was like to be significant. The functional enrichment of modules was performed with REACTOME.db pathway database incorporated in this tool for comprehensive biological illustration of the network.

### Quantitative real-time PCR (qRT-PCR)

For technical validation of microarray results, five selected DEGs (Table 1) known to be involved in immune response function were quantified by qRT-PCR in the same RNA samples as used for microarray expression. Primers were designed based on an open source primer designing software Primer3 [41]. First Strand cDNA Synthesis Kit (P/N K1612, Thermo Scientific, Co.) was used for reverse transcription with oligo (dT) primer. The qRT-PCR reaction was set up taking 1.0 μl of cDNA template, 8.0 μl of deionized RNase free water, 0.5 μM of upstream and downstream primers, and 10 μl iTaq™ Universal SYBR® Green Supermix (Bio-Rad laboratories GmbH, Germany) in a total reaction volume of 20 μl and were amplified by the StepOnePlus™ Real-Time PCR System (Applied Biosystems®, Darmstadt, Germany). The thermal cycling conditions were 95 °C for 3 min, 95 °C for 15 s, 6 °C for 45 s (40 cycles); 95 °C for 15 s, 62 °C for 1 min, 95 °C for 15 s. All reactions were run in duplicate and the average value was used for calculating the expression value. Gene-specific expression was measured as relative to the geometric mean of the expression of two housekeeping genes (GAPDH and ACTB) (Table 1). The delta delta Ct (∆∆Ct) method was used for calculating the difference between target gene and reference genes [42]. The correlation between microarray and qRT-PCR results was analyzed by Spearman’s Rho test. The significance level was set as *p* < 0.01.

**Table 1:** Primer sequences for used qRT-PCR validation. List of primers and their sequences of selected candidate genes used for qRT-PCR validation of microarray data

GenBank	Gene name	Primer sequence (5′-3′)
Accession number		
NM_213770.1	IRF3: Interferon regulatory factor 3	F: CCAGTGGTGCCTACACTCCT
		R: AGAGGTGTCTGGCTCAGGAA
NM_001044580	STAT3 : Signal transducer and activator of transcription 3 (acute-phase response factor)	F: TGCTGGAGGAGAGAATCGT
		R: GGGAATTTGACCAGCAATC
NM_214087	CD80: Cluster of differentialtion-80	F: TCAGACACCCAGGTACACCA
		R: GACACATGGCTTCTGCTTGA
NM_001105286.1	TRAF6: Tumor necrosis factor receptor-associated factor	F: GGGAACGATACGCCTTACAA
		R: CTCTGTCTTAGGGCGTCCAG
NM_213779	CCL4 : Chemokine (C-C motif) ligand 4	F: CTCTCCTCCAGCAAGACCAT
		R: CAGAGGCTGCTGGTCTCATA
HQ013301	GAPDH : Glyceraldehyde-3-phosphate dehydrogenase^a^	F: GCTGGTGCTGAGTATGTCGT
		R: AAGCAGTTGGTGGTACAGG
XM_003124280.3	ACTB: Actin, Beta^a^	F: AAGGACCTCTACGCCAACAC
		R: CTGGCTGATCCACATCTGCT

^a^ are the house keeping genes used for normalization

## Results

### PRRSV-specific antibody responses

**Fig. 1:**
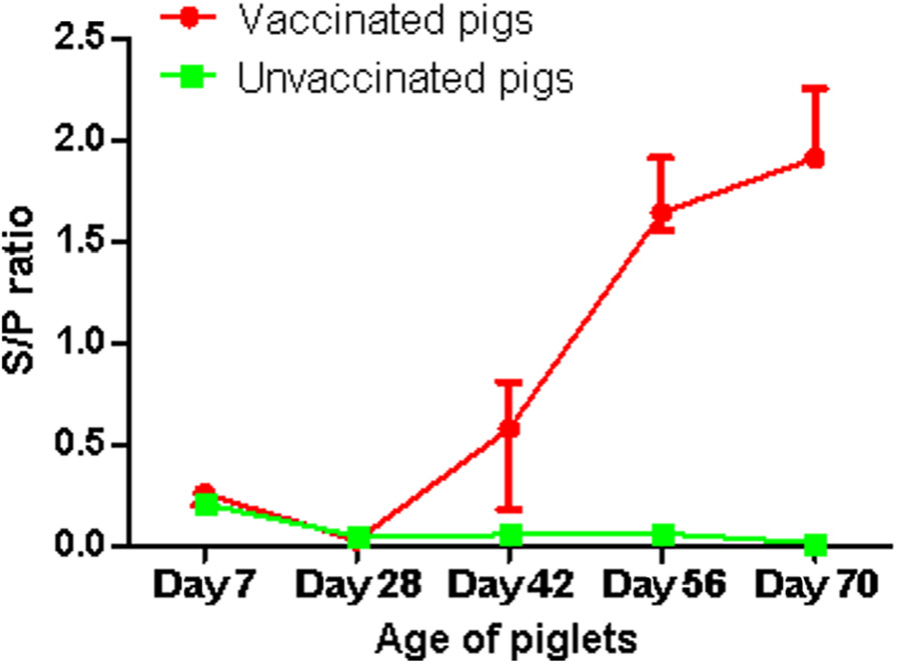
PRRSV-specific antibody responses. The figure depicts the reactivity of maternally derived antibody and vaccine derived antibody to PRRSV in plasma detected by PRRSV-AK Enzyme Immunoassay. Values in the Y-axis represent the sample to positive (s/p) ratio, and the s/p values of 0.4 was considered as threshold to classify the individuals either positive or negative. Values in X-axis represents the piglet ages at which blood samples were evaluated. Primary and booster vaccination were performed at day 28 and 56 of age, respectively in pigs of vaccinated group. In vaccinated group, the optical density (OD) values of samples at day 7 and 28 of age represent for maternally derived antibody (MDA), and samples at day 42, 56 and 70 for vaccine induced antibody response. While samples from the control group were used for only monitoring the way of declining the MDA over the age of animals in absence of further PRRSV exposure

In order to exclude the maternally derived antibody (MDA) response of PRRSV as well as to evaluate the vaccine induced antibody response, plasma samples from all pigs at day 7, 28, 42, 56 and 70 of age were screened by ELISA. The plasma antibody level confirmed that experimental pigs were negative for MDA of PRRSV considering the sample to positive (s/p) ratio of 0.4 (40 %) as threshold (Fig. 1). The optical density (OD) values indicated a relatively higher MDA titre in suckling piglets which felt down and remained stable towards the base line along with increased age of unvaccinated pigs. On the other hand, there was an increasing trend of plasma antibody titre in pigs following vaccination. The antibody titre got above the threshold after 2 weeks, and subsequently reached a plateau after 4 weeks of the primary vaccination (Fig. 1).

### Transcriptome profiles of PBMCs following PRRSV vaccination

To uncover the transcriptional modification underlying the innate immune response to a live attenuated PRRSV vaccine, we performed the global transcriptome profiling of PBMCs from pigs of vaccinated group at before (control) and 6, 24 and 72 h after vaccination; and from unvaccinated group at 6, 24 and 72 h post vaccination time points using the Affymetrix GeneChip Porcine Gene 1.0 ST Array. This array was encoded with 394,580 probe (20–22 probes per gene) representing a total of 19,212 known genes. After normalization the current study identified a total of 27,558 probes having higher signal intensity than the background. After filtering, 14,231 transcripts were found to be expressed in PBMCs, 10,217 of which could be annotated and were implemented in the downstream analyses.

### Variation of PBMCs transcriptome profiles between vaccinated and unvaccinated pigs

The gene set enrichment analysis (GSEA)-based comparison of genome-wide expression profiles has distinguished the vaccine induced transcriptome changes between the vaccinated pigs and the age-matched unvaccinated control pigs. The GSEA algorithm revealed that a total of 42 and 36 gene sets (pathways) were significantly upregulated at 6 and 24 hpv in vaccinated group, respectively, compared to their unvaccinated counterparts. Of these, the chemokine signaling, JAK-STAT signaling and cytoskeleton activation are the most significantly enriched gene sets which indicated the potential of vaccine to switch on the transcriptional machinery in PBMCs (Additional file 2). The enrichment score of most of the up regulated gene sets in the vaccinated group was increased at 24 hpv from that of 6 hpv indicating the number of core genes of particular gene set increased over the time of immunization (Table 2).

**Table 2:** Significantly enriched gene sets (pathway) obtained from gene set enrichment analysis. Top most gene sets enriched in PBMCs of PRRSV vaccinated pigs compared to unvaccinated control pigs exported from GSEA desktop application

Gene sets	6hpv_vacc vs. 6hpv_unvacc		24hpv_vacc vs. 24hpv_unvacc	
	NES	FDR	NES	FDR
Regulation of actin cytoskeleton	2.51	0.001	2.75	0.001
Chemokine signaling pathway	2.48	0.001	2.56	0.001
JAK-STAT signaling pathway	2.39	0.001	2.55	0.001
Integrin cell surface interactions	2.46	0.001	2.5	0.001
Cell adhesion molecules	2.31	0.001	2.38	0.001
Integrin signaling pathway	2.28	0.001	2.37	0.001
Cell surface interactions at the vascular wall	2.27	0.001	2.33	0.001
Signal transduction by L1	2.24	0.007	2.27	0.007
Cytokine cytokine receptor interaction	2.20	0.008	2.25	0.007
Apoptosis by serum deprivation up	2.17	0.008	2.23	0.007
Immortalized by HPV31 DN	2.16	0.008	2.23	0.008
Signaling by FGFR1 mutants	2.14	0.011	2.21	0.010
TNF signaling up	2.12	0.014	2.19	0.013
ECM receptor interaction	2.10	0.025	2.13	0.021
TRAF trafficking pathway	2.08	0.013	2.12	0.011
Leukocyte transendothelial migration	2.04	0.008	2.14	0.007

*NES* normalized enrichment score, *FDR* false discovery rate

### Differential gene expression in PBMCs after PRRSV vaccination

To get a comprehensive overview of transcriptional modifications associated with innate immune response, we performed the differential gene expression analysis over three time points (6, 24 and 72 hpv) after vaccination compared to the control (before vaccination). The normalized expression values for only main probes of the chip were included for differential expression analysis and filtered by the thresholds of FDR <0.01 and log_2_ fold-change >1.5 or < −1.5. Using this criterion, 2,453 transcripts were found to be differentially expressed in PBMCs after PRRSV vaccination. Among them, 1087 (44.31 %) gene transcripts could be annotated. A complete list of the differentially expressed genes (DEGs) in PBMCs at three time points following PRRSV vaccination is provided in Additional files 3, 4 and 5.

**Fig. 2:**
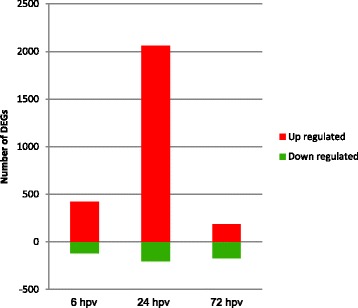
Number of differentially expressed genes after PRRSV vaccination. The figure depicts the number and direction of DEGs identified at three time points (6, 24 and 72 hpv) of PRRSV vaccination compared to the control (before vaccination)

The number DEGs and their direction of expression in three pairwise comparisons are plotted in Fig. 2. A total of 542 DEGs including 423 up regulated and 119 down regulated genes were detected at 6 hpv. The highest number (2, 263) of DEGs was identified at 24 h post vaccination. The number of upregulated genes (2,060) was also much higher than the down regulated ones (203) at 24 hpv. A total of 357 genes showed differential expression at 72 hpv in which 188 and 169 were up and down regulated, respectively. The fold change (FC) of differential expression ranged from −3.76 to 3.94; from −3.7 to 4.45 and from −4.15 to 3.11 at 6 hpv, 24 hpv and 72 hpv, respectively. A higher proportion of up regulated genes at each comparison indicated that vaccination induces active gene expression processes which may be associated with development of innate immune response.

**Fig. 3:**
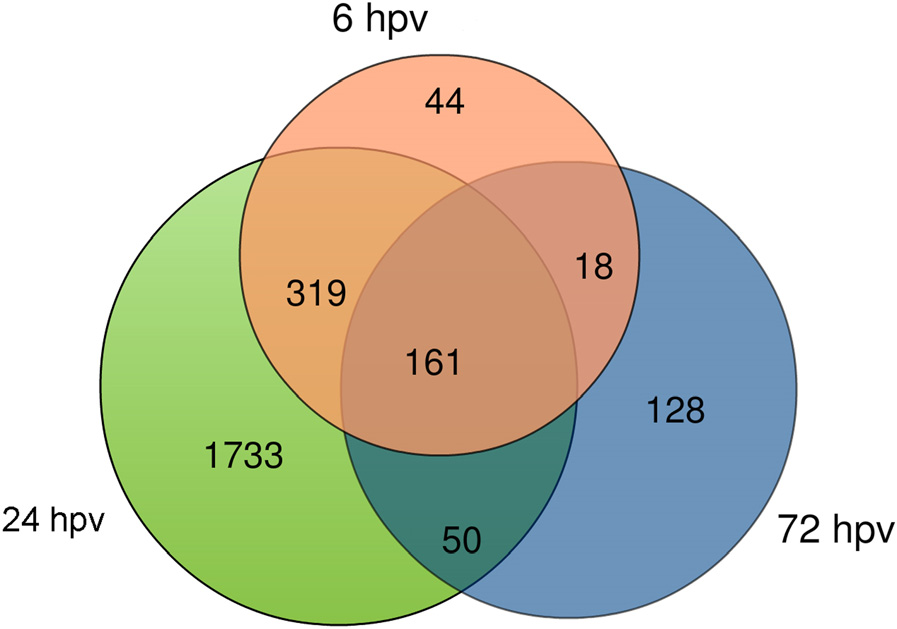
Intersecting Venn diagram showing the abundance of DEGs. The number of genes differentially expressed at three different time points (6, 24 and 72 hpv) of PRRSV vaccination compared to the control (before vaccination). The numbers in overlapping area(s) represent the differential expression of genes shared among the time points

The intersecting Venn diagram (Fig. 3) revealed that 44, 1,733 and 128 genes showing differential expression exclusively at 6, 24 and 72 hpv, respectively. Among the time point specific DEGs, 32, 1, 404 and 88 were up regulated and 12, 329 and 30 were down regulated at 6, 24 and 72 hpv, respectively. On the other hand, 161 genes showed differential expression constantly over the 3 days of post vaccination. Differential expression of 480 genes shared between the time points of 6 hpv and 24 hpv; 211 genes between 24 hpv and 72 hpv, and 179 genes shared between 6 hpv and 72 hpv time points.

**Fig. 4:**
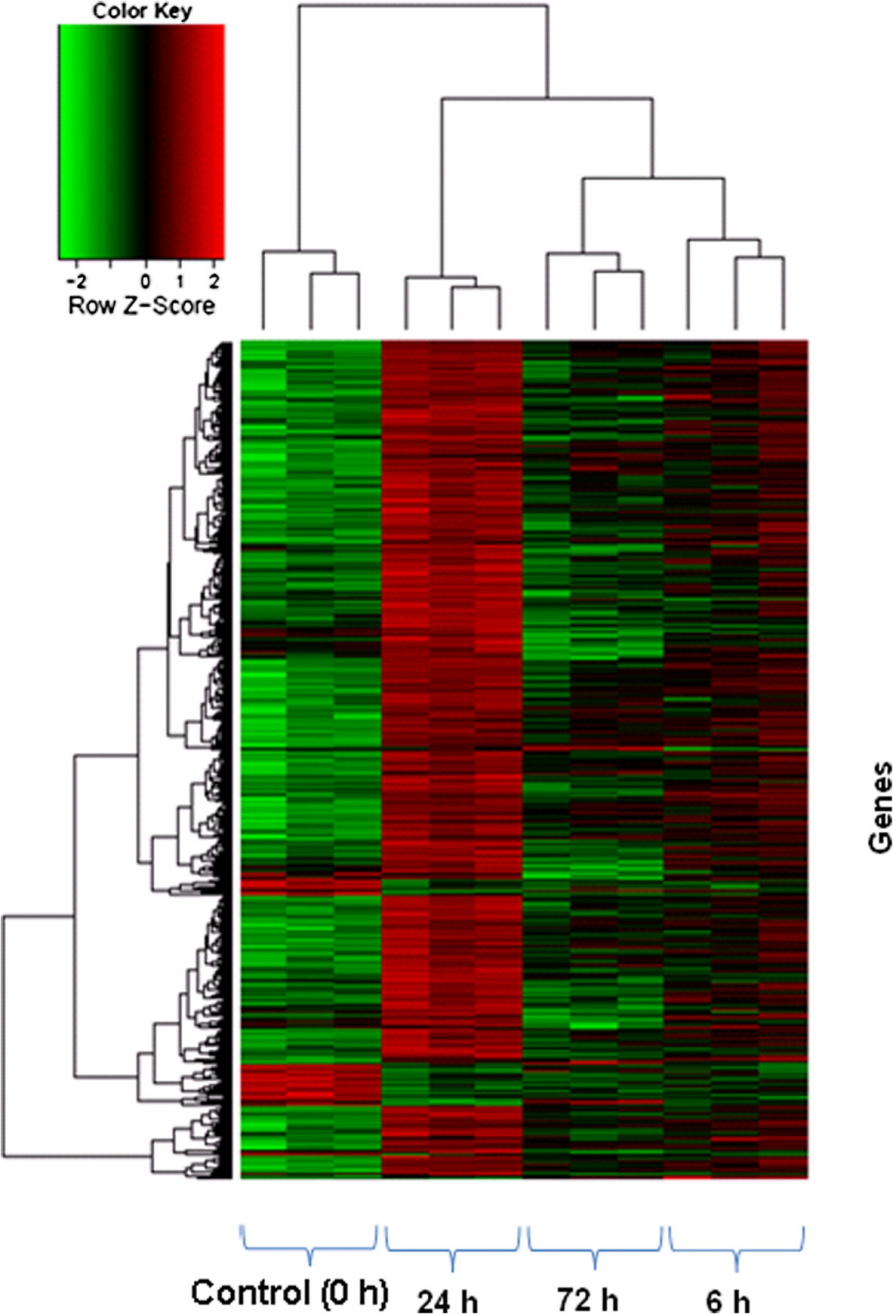
Hierarchical heat map showing differential gene expression over time. Normalized log_2_ transformed values as determined by Affymetrix GeneChip® porcine gene 1.0 ST array in PBMCs of German Landrace pigs at 6, 24 and 72 h post PRRSV vaccination. The cutoff value of log_2_ fold change as either ˃1.5 or ˂ −1.5 and FDR <0.05 was considered for statistical significance. Each column represents one array from each of replicate piglets

Hierarchical clustering of DEGs in PBMCs following vaccination has also provided a clear image of genes that were regulated in the same or opposite direction in response to vaccination (Fig. 4). There was distinction among time points of vaccine exposure in terms of up or down regulation of DEGs as well. A quite remarkable difference was observed at 24 h post vaccination compared to that of control. The hierarchical cluster analysis (HCA) indicates a good cluster of replicate piglets within the group which is suggestive for the homogeneity of the experimental blocks.

### GO and pathways enriched by PRRSV vaccine induced DEGs

**Fig. 5:**
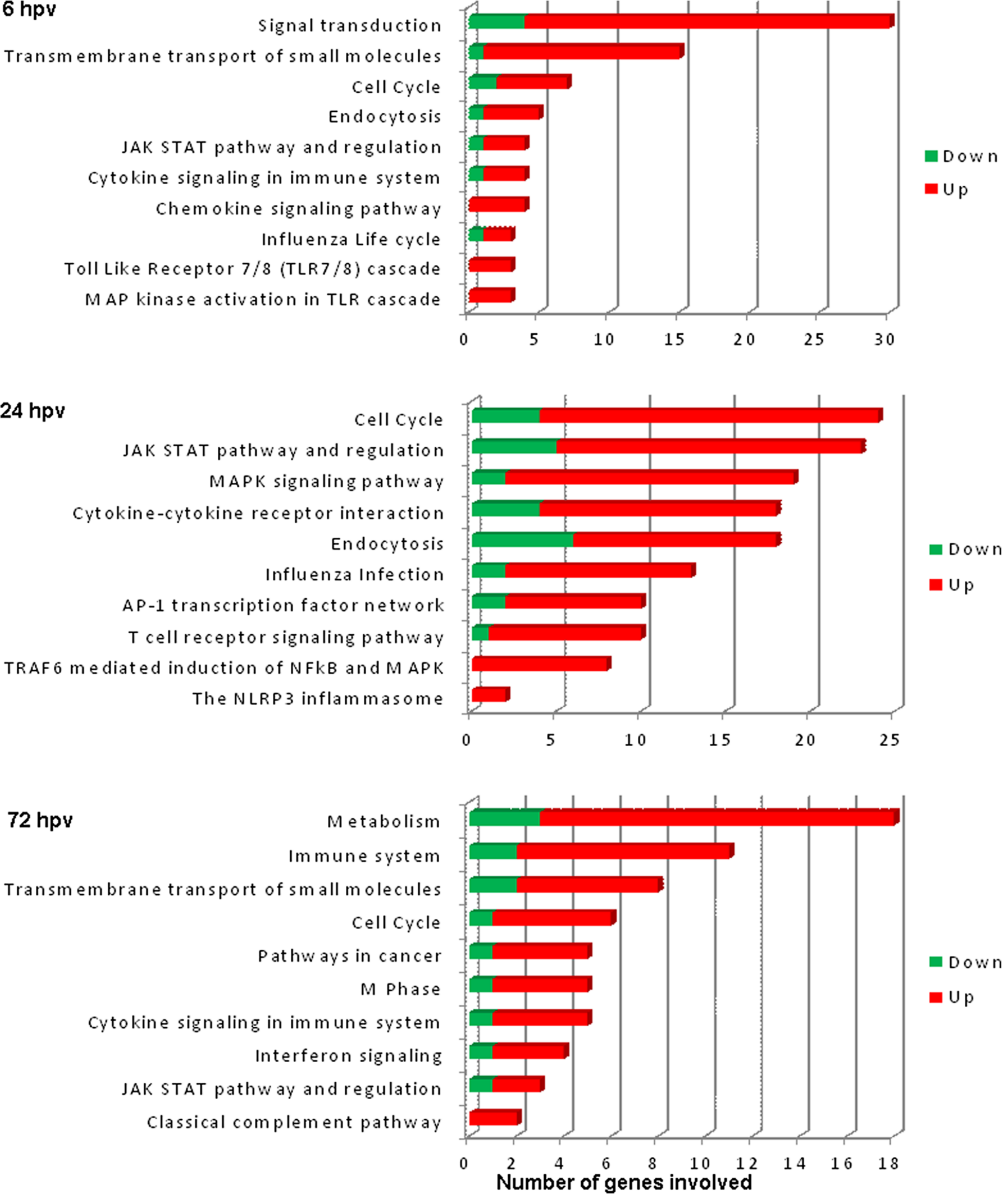
Biological pathways involved with the DEGs following PRRSV vaccination. The figure depicts the top ten biological pathways regulated by the DEGs in each of three pairwise comparisons. Values in X-axis represents the number of over-expressed genes (*red portion of bar*) and under-expressed genes (*green portion of bar*) involved in corresponding pathways. Pathways included here only having the over-representation *p* < 0.05 obtained from InnateDB. The upper part of graph (6 hpv) represents the top ten pathways regulated by DEGs found at 6 h, the middle (24 hpv) for that of 24 h and the bottom one (72 hpv) for that of 72 h of post vaccination time points compared to the control (before vaccination)

A GO classification of biological processes involved with all differentially expressed genes in PBMCs after PRRSV vaccination is provided in Table 3. The GO categories with a direct relation to immune response function includes innate immune response, signal transduction, viral process, T cell differentiation, chemotaxis, response to light stimulus, cytokine-mediated signaling pathway, complement activation, cell death, cell proliferation and immune system process. Highest representation of genes involved with particular GO terms was observed at 24 hpv compared to 6 hpv and 72 hpv. The pathway analysis paints a similar picture to the GO terms. The statistically significant biological pathways involved with PRRSV induced DEGs are presented in Fig. 5. Among the top pathways, *cytokine-cytokine receptor interaction*, *chemokine signaling pathway*, s*ignal transduction*, *JAK-STAT pathway and regulation*, *TRAF6 mediated induction of NF-kB and MAPK*, *the NLRP3 inflammasome, endocytosis and interferon signaling* were activated. The majority of genes involved in corresponding pathways were up regulated after vaccination.

**Table 3:** Gene ontology terms enriched by the DEGs. Top 30 GO terms of biological process involved with the vaccine induced DEGs retrieved from InnateDB pathway analysis tool. The significance of over-representation was defined by multiple test corrected *p*-value of <0.05

Time points	GO ID	GO term	Nr. of genes involved	Adjusted *p*-value
6 hpv	GO:0045087	Innate immune response	18	0.01
	GO:0042493	Response to drug	9	0.05
	GO:0034097	Response to cytokine	5	0.02
	GO:0016567	Protein ubiquitination	8	0.01
	GO:0043687	Post-translational protein modification	7	0.01
	GO:0044281	Small molecules metabolic process	30	0.01
	GO:0015031	Protein transport	10	0.03
	GO:0006355	Regulation of transcription, DNA-template	28	0.03
	GO:0007186	G-protein coupled receptor signaling pathway	15	0.03
	GO:0044267	Cellular protein metabolic process	14	0.03
24 hpv	GO:0045087	Innate immune response	90	0.01
	GO:0007165	Signal transduction	82	0.01
	GO:0008284	Positive regulation of cell proliferation	45	0.02
	GO:0016032	Viral process	37	0.01
	GO:0051607	Defense response to virus	14	0.02
	GO:0043406	Positive regulation of MAPK kinase activity	9	0.05
	GO:0006874	Cellular calcium ion homeostasis	11	0.08
	GO:0007265	Ras protein signal transduction	8	0.02
	GO:0030217	T cell differentiation	5	0.05
	GO:0035666	TRIF-dependent toll-like receptor signaling pathway	8	0.05
72 hpv	GO:0043408	Regulation of MAPK cascade	7	0.04
	GO:0007067	Mitotic nuclear division	6	0.04
	GO:0019221	Cytokine-mediated signaling pathway	5	0.03
	GO:0006935	Chemotaxis	4	0.04
	GO:0007155	Cell adhesion	7	0.05
	GO:0051726	Regulation of cell cycle	9	0.05
	GO:0055085	Transmembrane transport	14	0.03
	GO:0010467	Gene expression	6	0.05
	GO:0006915	Apoptotic process	8	0.04
	GO:0009615	Response to virus	5	0.14

### Transcriptional network of PRRSV vaccine induced innate immune responses in PBMCs

**Fig. 6:**
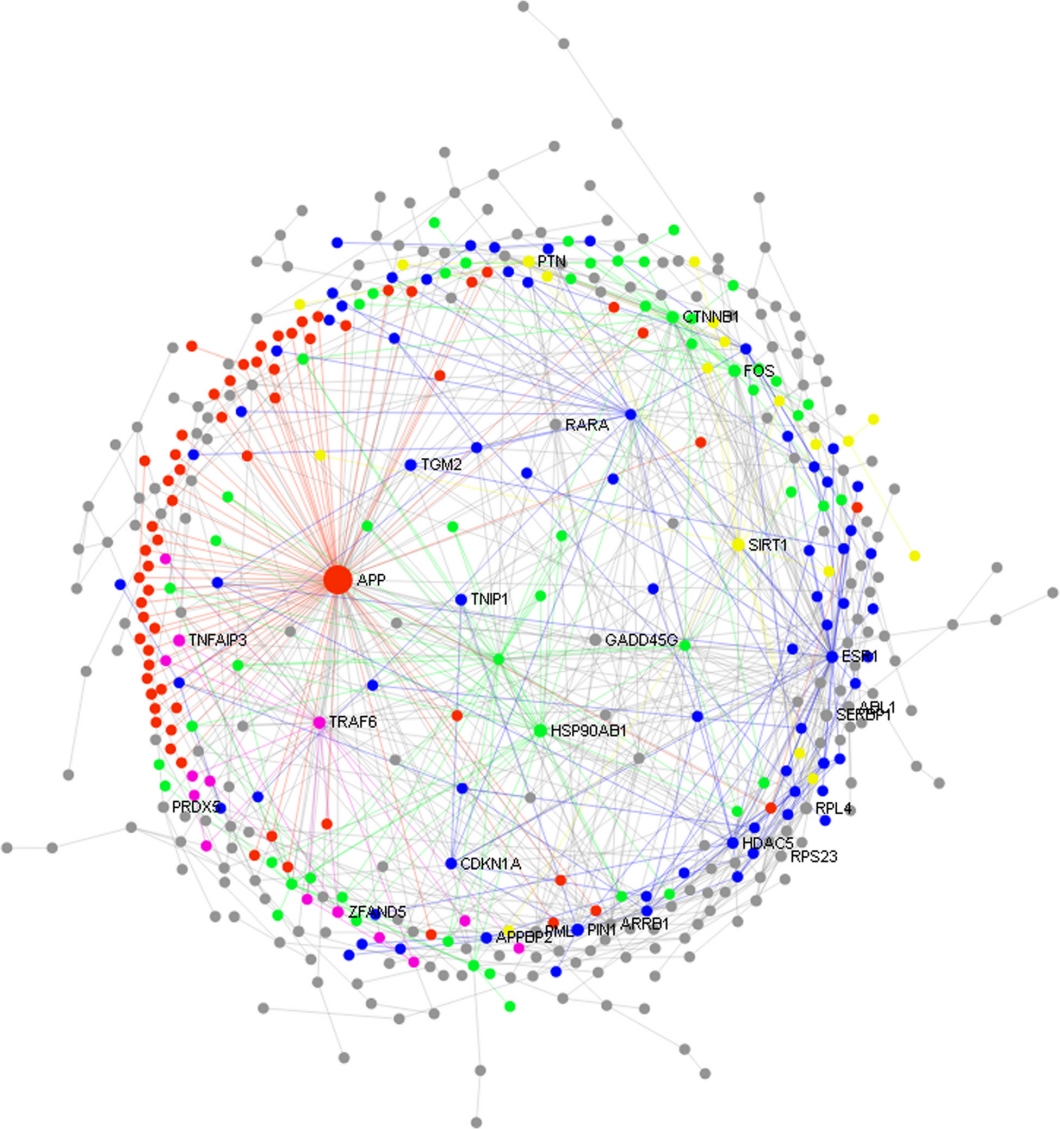
Global transcriptional network of PRRSV vaccine response in PBMCs. The picture depicts the interconnected network of PRRSV vaccine induced differentially expressed genes in PBMCs at 24 hpv. Each *circle* indicates the node or member genes of the network. The diameter of the circle corresponds to the values of two centrality measures that is degree and betweenness of particular gene. The larger diameter indicates the higher potential of the nodes to be the hub genes of the network. The network modules with corresponding genes are indicated by different colors

The network analysis retrieved one giant subnetwork herein called the global network and 12 other smaller networks. The global network was comprised of 432 seed genes or nodes and 850 edges or connections. The diameter of each node corresponds to the values of two centrality measures (degree and betweenness) and thereby a larger diameter indicates higher potential of particular node to be a hub of the network (Fig. 6). The values of degree and betweenness centrality of all seed genes are presented in Additional file 6. Based on these two centrality measures, *APP* (Amyloid beta (A4) precursor protein) was determined to be the top most potential hub gene of the global network having highest values of degree (118) and betweenness centrality (6468). Other potential hubs includes *TRAF6* (*TNF* receptor-associated factor 6), *PIN1* (Peptidyl-prolyl cis-trans isomerase NIMA-interacting 1), *FOS* (FBJ murine osteosarcoma viral oncogene homolog), *CTNNB1* (Catenin (cadherin-associated protein) beta 1), *CDKN1A* (Cyclin-dependent kinase inhibitor 1A (P21, Cip1)-A), *TNFAIP3* (Tumor necrosis factor, alpha-induced protein 3), *SIRT1* (Sirtuin 1), *ESR1* (Estrogen receptor 1) and *HDAC5* (Histone deacetylase 5).

The NetworkAnalyst tool has detected five significant (*p* <0.01) network modules within the global network which are indicated by different colors (Fig. 6). Each module was led by one or more of the above mentioned hubs connected too many other genes of similar biological function. Functional enrichment of the modules revealed that PRRSV vaccine induced transcriptional modification involves five major groups of biological functions such as innate immune response; development and differentiation of blood cell; cell death, cell cycle and survival; ubiquitination and glycosylation; and protein metabolism and regulation of gene expression. In particular, the functional involvement of the *APP* led network module (red module) includes membrane trafficking (*RAB5C, ARRB1, SEC24C, TBC1D1*), chemokine receptor binding (*CXCR2, CXCL16*), interferon signaling (*IFNA8, IFNW1*), post-translational protein modification (*PIGO, GALNT12, MPI, SEC24C*) and asparagine N-linkage glycosylation (*MPI, SEC24C*).

**Fig. 7:**
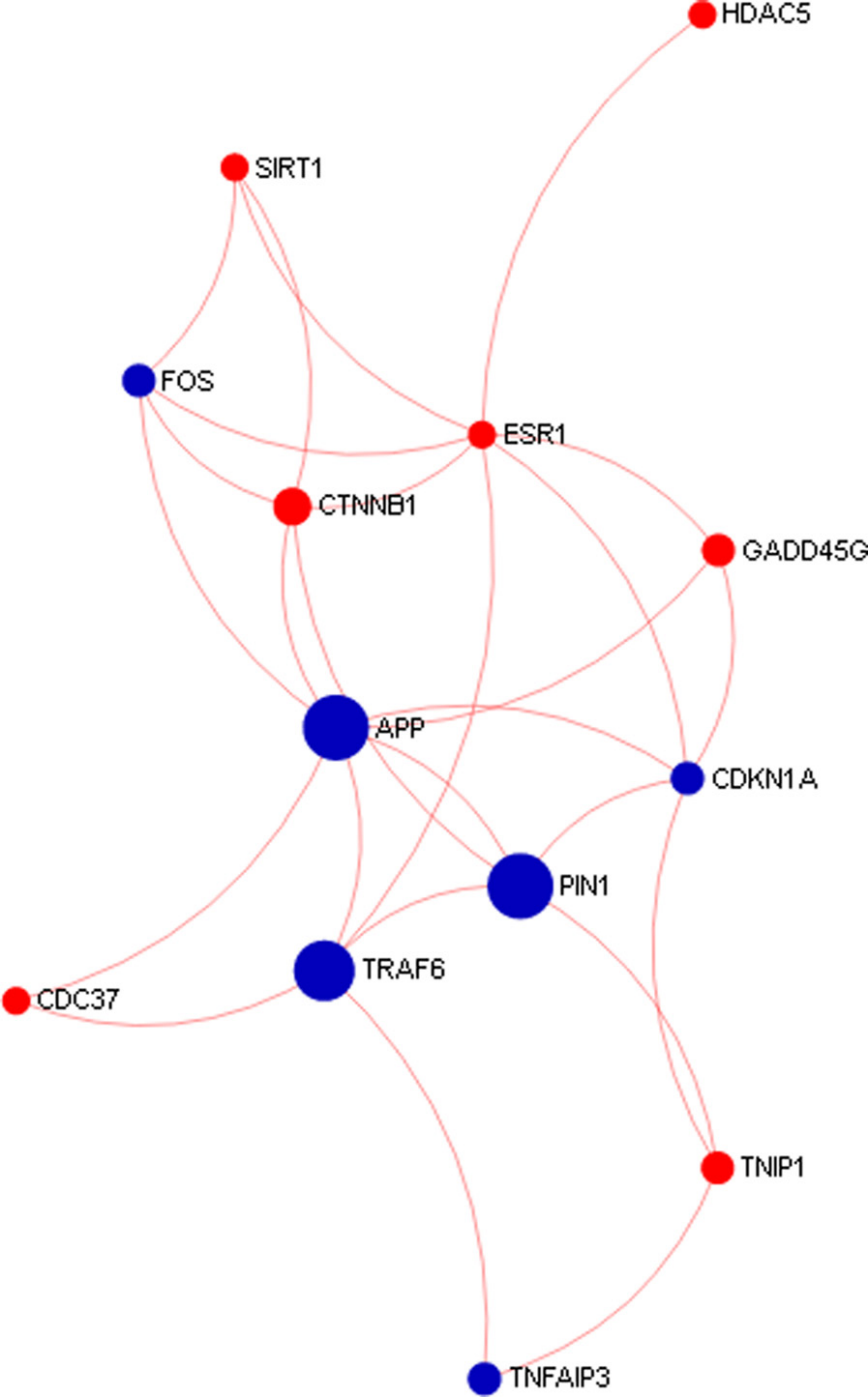
Core transcriptional network of PRRSV vaccine response in PBMCs. The picture depicts the connection among the regulatory genes of the global transcriptional network of PRRSV vaccine response in PBMCs. Only the direct connections among seed genes are presented. The diameter of the circle corresponds to the values of two centrality measures that is degree and betweenness of particular gene. Among the hubs, nodes of blue colors were known to be strongly involved in innate immune response function

To test whether these hub genes can coordinate the global transcriptional network, we constructed the second network (herein called the core network) taking the top thirteen hubs of global network as seed genes. The higher-order interactions of the core network assembled about 3,764 nodes and 5, 145 connections which reflected the global network. The simplified interconnection among the hub genes is presented in the core network (Fig. 7). Among the hub nodes, six (*APP, TRAF6, PIN1, FOS, CDKN1A* and *TNFAIP3*) were found to be directly involved with innate immune system and were upregulated in immunized PBMCs. *APP, TRAF6, PIN1, FOS* and *TNFAIP3* have direct connection with six, five, five, four and three other hubs, respectively. *TRAF, APP, CTNNB1, ESR1* and *HDAC5* together are responsible signal transduction process. *TRAF6* along with *FOS* participate in toll-like receptor cascades, *MAPK* signaling, *MyD88* dependent & independent cascades and proinflammatory response. *TRAF6* and *PIN* are involved in *RIG-1/MDA5* mediated induction of alpha-beta interferon.

### Validation of microarray data

**Fig. 8:**
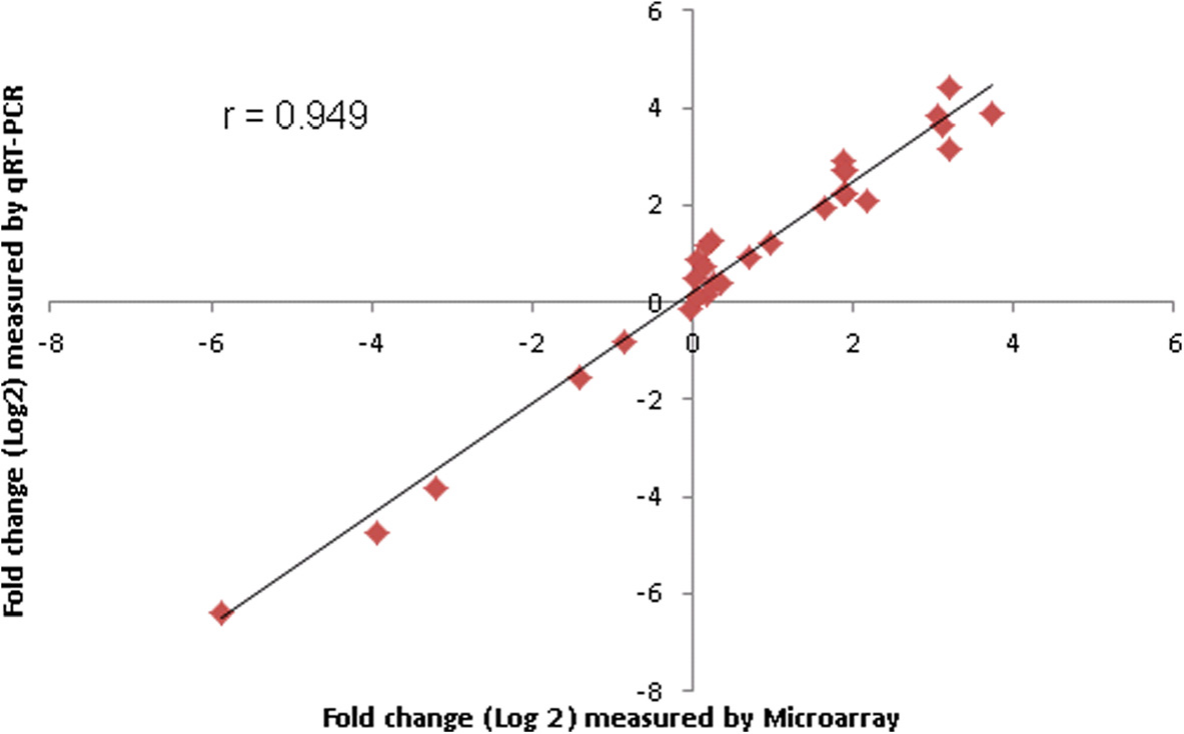
The qRT-PCR validation of the microarray data. The picture depicts the correlation between microarray (X-axis) and qRT-PCR (Y-axis) expression data (Log_2_ fold-change) for five selected genes at three different time points (6, 24, 24 hpv) both in vaccinated and unvaccinated pigs. Correlation between microarray and qRT-PCR data was analyzed by Spearman’s Rho test. The correlation coefficient was = 0.949, with a statistical significance of *p* < 0.01

Microarray data was validated through measuring the relative expression level of five differentially expressed genes (*STAT3*, *IRF3*, *CD80*, *CCL4* and *TRAF6*) in PBMCs using qRT-PCR. The expression data (Additional file 7) obtained from microarray and qRT-PCR for the selected genes are plotted in Fig. 8. The qRT-PCR expression values of all five genes confirmed statistically significant (*p* < 0.01) differential expression in the same direction as the microarray data with a correlation (correlation coefficient, *r* = 0.949).

## Discussion

Protective immunity to PRRS virus is a complex and unresolved issue. To date, the live attenuated virus vaccine has been considered to be the most economic method to achieve immunity and protecting herds from losses associated with infections by highly virulent strains of PRRSV [43]. In the current study, the antibody response appeared to start at 2 weeks of primary vaccination and reached a steady state at 4 weeks after primary vaccination in pigs (Fig. 1). This reflects the previous reports stating that PRRSV specific antibodies begin to appear in the infected pigs as early as 7 –10 days post infection with a low titre [44] followed by delayed production of neutralizing antibody (NAb) between 2 and 4 weeks post infection [44]. Besides neutralizing antibody, components of innate and cell mediated immune responses have major contribution to the viral clearance in immunized animals. Moreover, the character of the innate immune response to virus is thought to dictate the quality of subsequent adaptive immune response [45]. Therefore, we focused our transcriptome analyses on the innate immune response to vaccination.

To investigate the host-vaccine interaction, we generated the whole transcriptome profiles of PBMCs following in-vivo PRRSV (EU strain) vaccination in German Landrace pigs so far for the first time. Although some authors conducted gene expression studies on porcine PBMCs to evaluate the immune response to PRRSV [46, 47], but these were focused to in-vitro model with expression profiling of selected candidate genes. PBMCs are heterogeneous population of immune cells including monocytes, lymphocytes (T Cells, B cells, and NK cells) and dendritic cells. The frequency of proportion of PBMC-subpopulation may vary across individuals [48]. The vaccine induced cellular activation and differentiation may changes the proportion of sub-types of PBMCs, which are likely contribute to gene expression changes [49]. Thus, the current analyses have limitation in evaluating the cell type specific contribution on vaccine responses. In fact, the reports on, and option for, specific cell subset are limited in swine and mostly due to the relative lack of immune-tagged reagents critical for such detail phenotyping (reviwed by Schroyen and Tuggle [50]). However, specific cell type contribution could be partially addressed by bioinformatics approach of gene expression deconvolution. Indeed, the unfractionated PBMCs samples were analyzed in this study as a rapid and convenient model for monitoring the host transcriptional response to PRRSV vaccination.

With the global PBMCs transcriptome profiles, we first performed an exploratory functional analysis to characterize the phenotypic groups using GSEA algorithm. The GSEA first ranks all genes expressed based on the correlation (positive or negative) of their expression values with one of two phenotypes tested, then seeks the significance of over-representation of pre-defined gene sets (pathways) with the ranked gene list [36]. By this way, GSEA focused on identifying the pathways, not the individual genes differentially expressed between two contrast phenotypic groups. The GSEA-based analysis revealed significant enrichment of immunity related gene sets including chemokine signaling, JAK-STAT signaling and cytoskeleton activation (Additional file 2) in vaccinated PBMCs compared to that of unvaccinated one, which indicated the PRRSV vaccine potential to enhance the host’s innate immunity. This was consistence with the findings of a meta-analysis done by Badaoui et al. [51] who reported that the host-specific response to PRRSV challenge would be associated with the activation of canonical pathways like TREM1, toll-like receptor and hyper-cytokinemia/hyper-chemokinemia signaling. In addition, the hybridization values of selected cell surface marker genes including CD4, CD14, CD19, CD33 and CD86 (Additional file 8) indicated the changes of the proportion of PBMC-subpopulation following vaccination. This difference also justified the application of GSEA to distinguish the transcriptional responses between vaccinated and unvaccinated pigs through highlighting only those pathways important in the mechanism of innate immunity.

Followed by the gene set enrichment analysis, we performed the differential gene expression analyses in a time-series contrast which revealed that transcriptome alteration started at 6 hpv and peaked at 24 hpv followed by a decreased abundance at 72 hpv (Fig. 3). The differential expressions of five selective genes were confirmed by qRT-PCR (Fig. 8). It is noteworthy that the comparison between 6 and 24 h time points in unvaccinated group with that of 0 h in vaccinated group yielded about ~20 differentially expressed genes (data not shown) which were almost identical in both contrast pairs and did not lead to enrichment of any known immune response pathways. That indicated there was no significant variation among pigs having no vaccine exposures. Therefore, before vaccination (0 h) time point were used as control to compare with post vaccination time points. This was also supported by a similar study [27] where the pre vaccination samples have been used as control to investigate the temporal pattern of transcriptomic response in porcine PBMCs to Tetanus toxoid vaccine. The proportion of up regulated genes was much higher than the down regulated genes at all three time points indicated the potential of PRRSV vaccine to induce gene expression in PBMCs. The differential gene expression analysis of the present study showed massive changes in the transcript abundance of known immune response genes and of genes that have been implicated in PRRSV infection by several authors [3, 52, 53]. Xiao et al. [3] reported that 4,520 genes were differentially expressed in porcine lungs at 96 and 168 h after in-vivo infection with highly pathogenic PRRSV strain, and those altered genes were functionally linked to host innate immune responses.

The InnateDB pathway analyses of DEGs revealed that transcriptome modification caused by vaccination are involved with activation of pathway such as toll like receptor 7/8 cascade, endocytosis, cytokine signaling, chemokine signaling, signal transduction, *MAPK* activation in *TLR* cascade and *JAK/STAT* signaling pathway. These pathways are known to be involved in the process of host cell sensing of the viral antigen and subsequent induction of innate immune response. Innate immunity against viral antigen is initiated once after sensing of viral pathogen-associated molecular patterns (PAMPs) by the specific host cell pattern recognition receptors (PRRs) such as toll-like receptors 3, 7 and 8 (*TLR3*, *TLR7* and *TLR8*) [54]. We observed the up regulation of *TLR3* and *TLR7* in PBMCs after vaccination and genes of signal transducers and activators of transcription (*STAT*) family. The recognition of viral PAMPs (ss RNA for PRRSV) by the host cell TLR results in a cascade of intracellular signaling through various adapter molecules (e.g. *MyD88*, *MDA5, TRAF6*) followed by activation of the MAP kinase family, which in turn switch on transcription factors such as interferon regulatory factors (*IRFs*) and *NF-kB* [54]. Among the IRF family members, *IRF2*, *IRF2BPL*, *IRF5* and *IRF7* were up regulated but IRF3 was down regulated in PBMCs after vaccination. It has been reported that the members of *IRF* family such as *IRF3*, *ISGF3*, *ISG15*, *IKKα*, *STAT1*/*STAT2* are involved in immunosuppressive effects of PRRSV infected cells [55, 56]. The *NF-kB* induces several downstream signaling leading to the up regulation of proinflammatory cytokines, chemokines and type-I interferon which in turn facilitate the inflammatory process, apoptosis and phagocytosis [57], which are key cellular process of innate immunity.

Finally we performed the network analysis to extract the regulatory molecules for vaccine responses. The network analysis revealed that genes including *APP, TRAF6, PIN1, FOS, CDKN1A, CTNNB1, TNFAIP3 SIRT1, ESR1* and *HDAC5* are the most highly interconnected hubs of vaccine induced transcriptional network in PBMCs (Fig. 6). The common feature of these master switch genes is that they regulate the induction of several pathways of the innate immune responses including *TLR* signaling, *MAPK* kinase cascades, interferon signaling and advanced glycosylation endpoint receptor signaling. This is in line with the recent report on detection of network module containing numerous immune response genes through weighted gene co-expression network analysis of whole blood transcriptome profiles of PRRSV infected pigs [58]. Amyloid beta (A4) precursor protein (*APP*), the top hub gene, is a protein coding gene which induces the secretion of a number of peptides; two of the peptides were shown to have antibacterial and antifungal activities [59]. The *APP* has been reported to be over expressed in porcine alveolar macrophages 24 h after in-vitro stimulation with PRRSV [60]. The network module led by *APP* has functional involvement with asparagine N-linked glycosylation of surface glycoprotein 3 (GP3) of PRRSV which regulates the neutralizing antibody response [61]. Therefore, the *APP* led gene network module might contribute to PRRSV vaccine induced transcriptional responses in PBMCs.

*TNF* receptor-associated factor 6 (*TRAF6*) was found to be another prominent hub gene for transcriptional network induced by PRRSV vaccination in PBMCs. The *TRAF6* is an adapter molecules required for TLR (TLR7/8) induced signal transduction leading to expression of *IFNs* [62]. The peptidyl-prolyl cis-trans isomerase-1 (*PIN1*) is a nucleus protein which has an essential role in toll-like receptor signaling and type-1 interferon mediated innate immunity. *TLR7* and *TLR9* activate the isomerase *PIN1* which subsequently activates the *IRAK1,* IRAK2 and *IRF7* and induces type I interferons [63]. It appeared that among the top network hubs, *APP, TRAF6* and *PIN1* are known to be involved in the interferon response, the most potential antiviral innate immunity. Both *TRAF6* and *PIN1* are located in *Sus scrofa* chromosome 2 (SCC2), where the QTL for interferon-gamma level has already been identified [64]. However, no QTL for immune response capacity have been reported yet on the SSC13 where *APP* is located. Another hub gene, *FOS,* is a nuclear phosphoprotein involved in signal transduction, cell proliferation and differentiation, has been reported to regulate the replication of hepatitis-c virus [65]. The cyclin-dependent kinase inhibitor 1A (CDKN1A) is a protein coding gene known to be involved in antiviral immune response in human [66]. Both *FOS* and *CDKN1A* are located on chromosome 7 (SSC7) where at least two QTLs for PRRS resistance as well as QTL for other innate immune response trait have been reported [67]. Tumor necrosis factor alpha-induced protein 3 (*TNFAIP3*) is also found as hub of the transcriptional network of PRRSV vaccine response in PBMCs. The *TNFAIP3* is a ubiquitin editing enzyme, known to be involved in inflammatory responses signaled by cytokines, such as *TNF*-alpha and *IL-1* beta, or pathogen sensing via toll-like receptors (TLRs) through terminating NF-kappa-B activity [47]. *TNFAIP3* is located on chromosome1 (SSC1) where at least three QTLs for PRRS susceptibility has been reported [19, 21, 67]. There were two close enzymatic products *SIR1* and *HDAC5* also found as hubs of the transcriptional network. *SIRT1* deacetylates a wide range of substrates, including p53, *NF-kB*, *FOXO* transcription factors, and PGC-1α, with roles in cellular processes ranging from energy metabolism to cell survival [68]. However, *HDAC5* had strong connection only with *ESR1* in the core network. The *ESR1* was also found to be in the list of top ten hub genes of the network which was over expressed in vaccinated PBMCs. *ESR1* along with *PRLR*, *FSHB*, *EPOR* and *RBP4* were reported to have significant association with swine reproductive traits [69]. Though reproductive failure is one of the major clinical outcomes of PRRSV infection in breeding sows, *ESR1* does not currently have known roles in innate immunity to PRRSV and warrants further investigation. On the whole, the hub genes including *APP, TRAF6, PIN1, FOS, CDKN1A* and *TNFAIP3* (Fig. 7), among others, were found to be highly interconnected to maintain the innate immune response function. Therefore, these six hub genes would coordinately be able to control the transcriptional network of PRRSV vaccine induced innate immune responses in PBMCs.

## Conclusions

Herein, we performed microarray-based transcriptome profiling to investigate genes, pathways and networks that may be involved in innate immune response of PBMCs to PRRSV vaccination in German Landrace pigs. This study identified *APP, TRAF6, PIN1, FOS, CDKN1A* and *TNFAIP3* as potential hub genes which could contribute to the functional network of PRRSV vaccine induced transcriptome alteration in PBMCs. Improvement of host genetic resistance has recently been considered as a prospective way for sustainable PRRS control. As direct measurement of disease resistance is very difficult, an indirect approach through identification of genomic marker associated with innate immune response to PRRSV vaccine is recommendable. Therefore, it would imply that hub genes of the functional network identified in this transcriptome analysis might be considered for future research to investigate their potential role in PRRS resistance in pigs. However, the genetic diversity among the pig breeds might contribute to the variation of PRRSV vaccine responsiveness. The correlation between early stage gene expression pattern and the antibody response of PRRSV vaccination could also be tested in larger pig population.

## Additional files

Additional file 1: Experimental design. The figure depicts experimental design and sampling schedule from PRRSV vaccinated and unvaccinated pigs used for this study. Vertical lines indicated the blood sampling time points over the age of pigs (days). Primary and booster vaccination were performed at day 28 and 56 of age, and blood was collected immediately before vaccine injection in those days. Blood samples collected at 0, 6, 24 and 72 h after primary vaccination from both group except 0 h in unvaccinated group used for whole transcriptome microarray study. Three biological replicates from both groups were used for microarray hybridization. The same RNA samples used for microarray were quantified by qRT-PCR for technical validation of microarray data. Blood samples collected from all pigs at day 7, 28, 42, 56 and day 70 of their age were used for ELISA based monitoring of PRRSV specific antibody response. (PNG 75 kb) Additional file 2: Characterization of phenotypic groups by GSEA. The figure depicts the comprehensive results of gene set enrichment analysis of our gene expression dataset against the curated gene set catalogue of “C7: immunological signature (Molecular signature database, v5.0, Cambridge, MA)”. Enrichment plots for the 3 gene set (pathways) upregulated both at 6 hpv and 24 hpv in vaccinated cohort compared to their unvaccinated counterparts are shown on the left side with the relative gene positions indicated by the straight lines (line plot) under each graph. Lines clustered to the left represent higher ranked genes in the ranked list. Expression profiles for a subset of genes (shaded in yellow in the line plots) contributing to core enrichment for each pathway are shown to the right as a heatmap. The heatmap compares subject-level gene expression in both vaccinated and control subjects. Gene expression is normalized for each row. Lower levels of expression are represented in shades of blue and higher expression in red. (PNG 184 kb) Additional file 3: List of DEGs in PBMCs of pigs at 6 hpv of PRRSV vaccination in pigs compared to control. (XLSX 57 kb) Additional file 4: List of DEGs in PBMCs of pigs at 24 hpv of PRRSV vaccination in pigs compared to control. (XLSX 226 kb) Additional file 5: List of DEGs in PBMCs of pigs at 72 hpv of PRRSV vaccination in pigs compared to control. (XLSX 41 kb) Additional file 6: Degree and betweenness centrality measures of the seed genes of the network. (CSV 7 kb) Additional file 7: Microarray and qRT- PCR expression values obtained for the five selected genes for the validation of microarray results. Two house keeping genes (GAPDH and ACTB) were used for normalization of the expression values. (PDF 20 kb) Additional file 8: Microarray-based expression profiles of selected cell surface markers, (cluster of differentiation (CD)) in PBMCs of vaccinated group (A), and unvaccinated group (B). (PDF 213 kb)
