# Supplementary figures and images for: Deciphering transcriptome profiles of peripheral blood mononuclear cells in response to PRRSV vaccination in pigs
Source: BMC Genomics. 2016 Aug 15;17:641. doi: 10.1186/s12864-016-2849-1 (PMC4986384; doi:10.1186/s12864-016-2849-1)

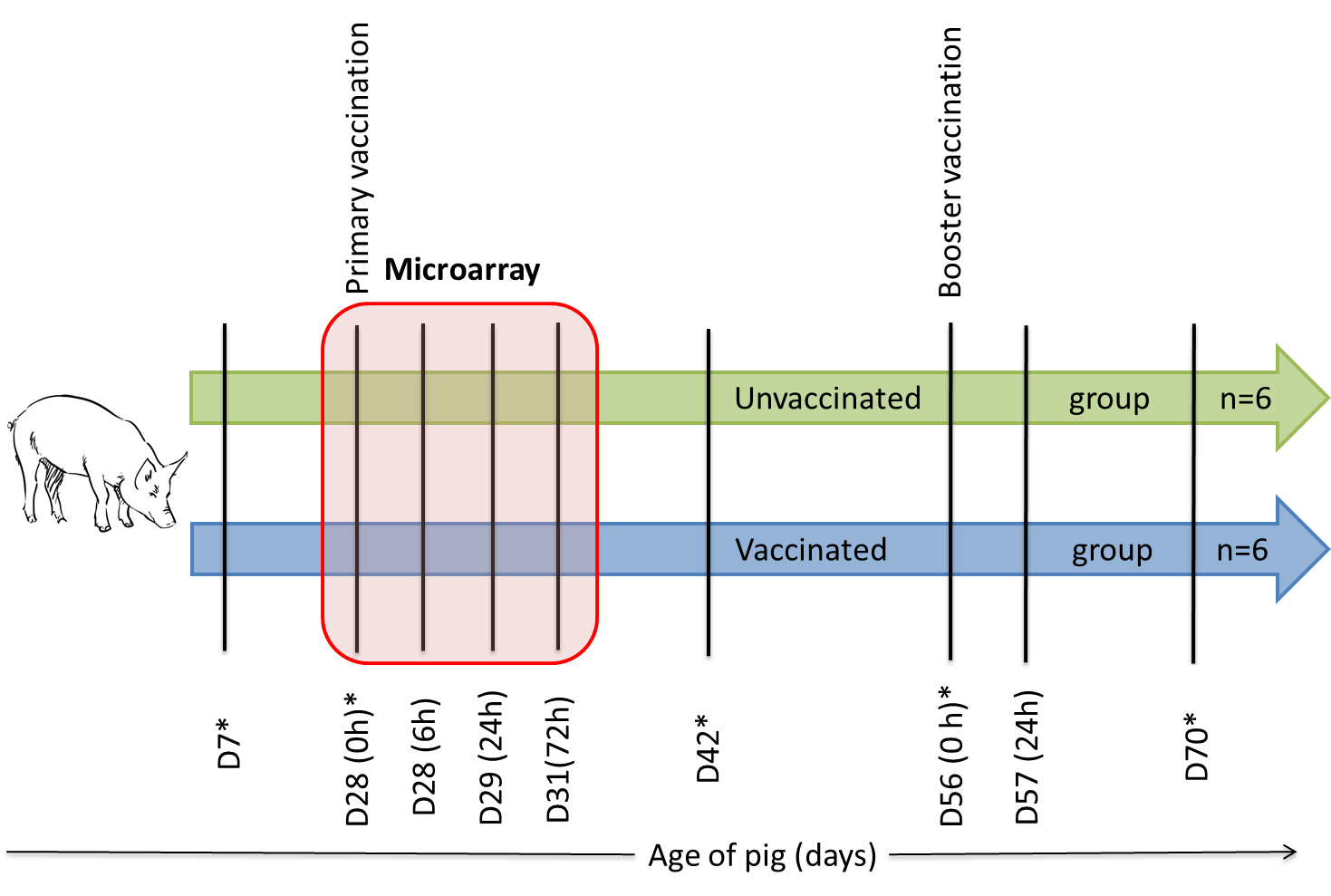

Supplement: Additional file 1: — Experimental design. The figure depicts experimental design and sampling schedule from PRRSV vaccinated and unvaccinated pigs used for this study. Vertical lines indicated the blood sampling time points over the age of pigs (days). Primary and booster vaccination were performed at day 28 and 56 of age, and blood was collected immediately before vaccine injection in those days. Blood samples collected at 0, 6, 24 and 72 h after primary vaccination from both group except 0 h in unvaccinated group used for whole transcriptome microarray study. Three biological replicates from both groups were used for microarray hybridization. The same RNA samples used for microarray were quantified by qRT-PCR for technical validation of microarray data. Blood samples collected from all pigs at day 7, 28, 42, 56 and day 70 of their age were used for ELISA based monitoring of PRRSV specific antibody response. (PNG 75 kb) [file 12864_2016_2849_MOESM1_ESM.png]

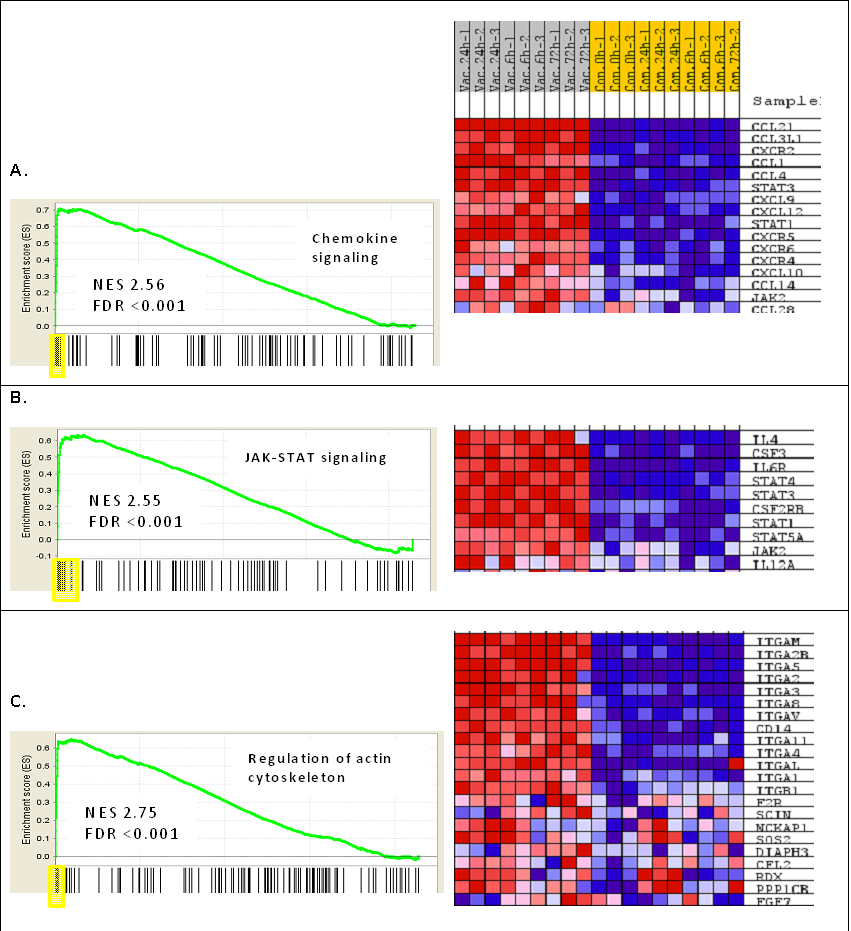

Supplement: Additional file 2: — Characterization of phenotypic groups by GSEA. The figure depicts the comprehensive results of gene set enrichment analysis of our gene expression dataset against the curated gene set catalogue of “C7: immunological signature (Molecular signature database, v5.0, Cambridge, MA)”. Enrichment plots for the 3 gene set (pathways) upregulated both at 6 hpv and 24 hpv in vaccinated cohort compared to their unvaccinated counterparts are shown on the left side with the relative gene positions indicated by the straight lines (line plot) under each graph. Lines clustered to the left represent higher ranked genes in the ranked list. Expression profiles for a subset of genes (shaded in yellow in the line plots) contributing to core enrichment for each pathway are shown to the right as a heatmap. The heatmap compares subject-level gene expression in both vaccinated and control subjects. Gene expression is normalized for each row. Lower levels of expression are represented in shades of blue and higher expression in red. (PNG 184 kb) [file 12864_2016_2849_MOESM2_ESM.png]

A.

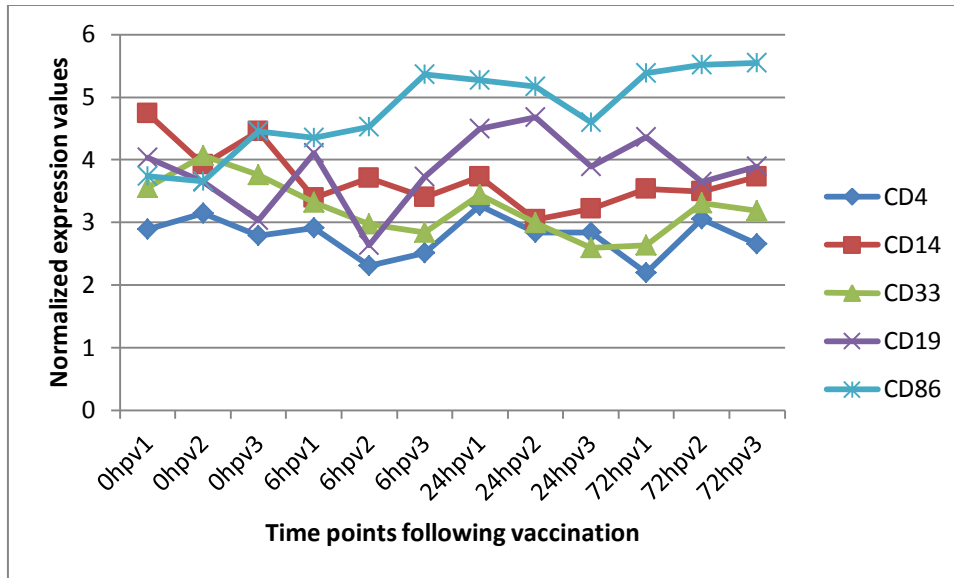

B.

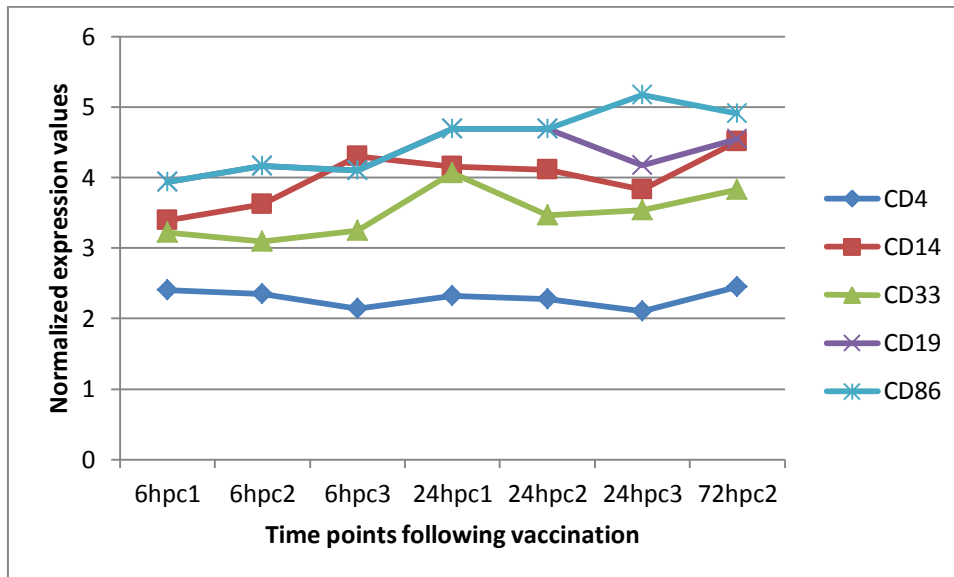

Supplement: Additional file 8: — Microarray-based expression profiles of selected cell surface markers, (cluster of differentiation (CD)) in PBMCs of vaccinated group (A), and unvaccinated group (B). (PDF 213 kb) [file 12864_2016_2849_MOESM8_ESM.pdf]
