# Supplementary material for: Deciphering transcriptome profiles of peripheral blood mononuclear cells in response to PRRSV vaccination in pigs
Source: BMC Genomics. 2016 Aug 15;17:641. doi: 10.1186/s12864-016-2849-1 (PMC4986384; doi:10.1186/s12864-016-2849-1)
Supplement: Additional file 7: — Microarray and qRT- PCR expression values obtained for the five selected genes for the validation of microarray results. Two house keeping genes (GAPDH and ACTB) were used for normalization of the expression values. (PDF 20 kb) [file 12864_2016_2849_MOESM7_ESM.pdf]

Genes	Treatment group	Time points	Fold change	
			Microarray	qRT-PCR
**STAT3**	Unvaccinated control	6h<br>24h<br>72h	0.71<br>0.96<br>0.27	0.90<br>1.22<br>0.41
	Vaccinated	6h<br>24h<br>72h	3.21<br>3.72<br>3.04	4.41<br>3.90<br>3.82
**IRF3**	Unvaccinated control	6h<br>24h<br>72h	-0.04<br>-0.84<br>-1.42	-0.12<br>-0.81<br>-1.53
	Vaccinated	6h<br>24h<br>72h	-3.21<br>-3.93<br>-5.88	-3.81<br>-4.73<br>-6.40
**CD80**	Unvaccinated control	6h<br>24h<br>72h	0.05<br>0.22<br>0.19	0.09<br>1.27<br>0.14
	Vaccinated	6h<br>24h<br>72h	1.88<br>2.17<br>1.92	2.9<br>2.10<br>2.21
**CCL4**	Unvaccinated control	6h<br>24h<br>72h	0.19<br>0.34<br>0.05	1.15<br>0.41<br>0.88
	Vaccinated	6h<br>24h<br>72h	3.12<br>3.21<br>1.88	3.62<br>3.15<br>2.71
**TRAF6**	Unvaccinated control	6h<br>24h<br>72h	0.04<br>0.14<br>0.06	0.51<br>0.72<br>0.11
	Vaccinated	6h<br>24h<br>72h	1.88<br>1.91<br>1.65	2.21<br>2.73<br>1.92
